# Chitosan Oligosaccharide Ameliorates Metabolic Syndrome Induced by Overnutrition *via* Altering Intestinal Microbiota

**DOI:** 10.3389/fnut.2021.743492

**Published:** 2021-10-01

**Authors:** Yihua Wang, Shili Liu, Di Tang, Rui Dong, Qiang Feng

**Affiliations:** ^1^School and Hospital of Stomatology and Shandong Provincial Key Laboratory of Oral Tissue Regeneration and Shandong Engineering Laboratory for Dental Materials and Oral Tissue Regeneration and School of Basic Medical Science, Cheeloo College of Medicine, Shandong University, Jinan, China; ^2^School of Mathematics, Shandong University, Jinan, China; ^3^State Key Laboratory of Microbial Technology, Shandong University, Qingdao, China

**Keywords:** chitosan oligosaccharides, metabolic syndrome, fecal microbiota, cecal microbiota, prebiotic effects

## Abstract

Chitosan oligosaccharides (COS) play a prebiotic role in many ways, whereas its function on microbiota is not fully understood. In this study, the effects of COS on metabolic syndrome were initially investigated by testing changes in the physiological indicators after adding COS to the diet of mice with high fat (group H) and low fat (group L). The results showed that COS markedly inhibited the accumulation of body weight and liver fat induced by high-fat diet, as well as restored the elevated concentration of blood glucose and fasting insulin to normal levels. Next, changes of the murine intestinal microbiota were examined. The results exhibited that COS reduced with-in-sample diversity, while the between-sample microbial diversity enhanced. Specifically, COS enriched *Clostridium paraputrificum* and *Clostridium ramosum* in the mice on a high-fat diet, while the abundance of *Clostridium cocleatum* was reduced. As a comparison, *Parabacteroides goldsteinii* and *Bacteroides uniformis* increased their abundance in response to COS in the low-fat diet group. Noticeably, a large amount of *Akkermansia muciniphila* was enriched in both high-fat or low-fat diet groups. Among the differential fecal bacteria, *Clostridium ramosume* was found to be positively interacted with *Faecalibacterim prausnitzii* and *Clostridium paraputrificum*; *Clostridium paraputrificum* had a positive interactions with *Lactococcus chungangensis* and *Bifidobacterium mongoliense*, suggesting that COS probably ameliorate metabolic syndrome through the microbiota in view of the lipid-lowering effects of these interacted bacteria. Furthermore, the gene expression data revealed that COS improved the functions related to intestinal barrier and glucose transport, which could be the trigger and consequence of the variations in gut microbiota induced by COS. Additionally, correlation analysis found that intestinal bacteria are related to physiological parameters, which further supports the mediating role of gut microbiota in the beneficial effect of COS. In summary, our research results provide new evidence for the prebiotic effects of COS.

## Introduction

Metabolic syndrome includes a group of metabolic risk factors, such as atherogenic dyslipidemia (elevated serum triglycerides, low high-density lipoprotein (HDL) cholesterol), enhanced blood pressure, and dysglycemia (insulin resistance and abnormal serum glucose), and the body is in a state of pro-inflammatory and pro-thrombosis (1, 2). When these risk factors are present at the same time, the occurrences of cardiovascular disease and type 2 diabetes are greatly promoted, and most people with metabolic syndrome are overweight. The most likely predisposing factor of metabolic syndrome is the accumulation of excessive lipids in organs or tissues caused by overnutrition; this in turn disrupts the metabolic process and leads to metabolic risk factors (3). Excess nutrition comes from either triglycerides or carbohydrates in the diet. Among them, dietary triglycerides enter the circulation with chylomicrons. Triglycerides are hydrolyzed into fatty acids by lipoprotein lipase (LPL); and most of the released fatty acids enter adipose tissue, where they are re-esterified into triglycerides. Glucose from dietary carbohydrates enters the tissues directly. When too much glucose is consumed, it can be converted into fatty acids through a process called adipogenesis. Tissue overload by lipid predisposes to metabolic syndrome (3). The prior interventions for overnutrition are dietary adjustments and more physical activity (4). Some drugs were used last decades as a choice of intervention, such as sibutramine, orlistat, phentermine, diethylpropion, and fluoxetine; but most of them have been withdrawn from the market owing to their high cost and serious long-term side effects (5, 6). There is an urgent need for safe and effective weight-loss drugs to treat overnutrition; natural compounds have become a good choice due to their high activity and few side effects.

The natural polysaccharide chitin is one of the most abundant biopolymers in natural environment (7), and it is N-acetyl-2-amino-2-deoxyglucose (GlcNAc) units linked by β-(1 → 4) bonds. Chitinolytic enzymes (chitinase and chitobiase) break down the glycosidic bonds between GlcNAc units and degrade chitin into chitosan. D-glucosamine oligomers (chitosan oligosaccharide, COS) is one of the main degradation product of chitosan/chitin (8). Due to molecular weight ≤16 kDa, COS is readily soluble in water and particularly useful for a series of industrial purposes. COS has already been proven to be effective in reducing body weight, lowering serum triglyceride and cholesterol levels, and limiting lipid accumulation in hepatocytes and adipose tissue (9, 10). The currently reported anti-obesity mechanisms of COS mainly include inhibition of apolipoprotein B (ApoB) levels, PPAR-γ, bile acid secretion, pancreatic lipase production and reduction of serum ghrelin concomitant with increasing leptin (8). However, the experiments to study the effect of COS are all short-term so far (11), and the beneficial effects and safety of COS on the body are still uncertain. Some negative effects have been reported, such as gastrointestinal discomfort and bloating/flatulence (12), which still need long-term experiment to verify the beneficial effects of COS. Moreover, more exploration is still needed to dig out more mechanisms, and the wide application of COS also requires satisfactory stability and safety assessment as a guarantee.

Disorder of intestinal microbiota has been recognized as an important cause of obesity in recent years, the intestinal bacteria are proved to be ideal targets for the treatment of obesity (13). For example, intestinal *Bacteroides thetaiotaomicron* may contribute to glutamate metabolism and attenuate obesity (14). Therefore, prebiotics have been widely used in recent years to adjust intestinal microbiota and improve obesity (15). Studies have proven that the potential beneficial effects of COS on host health are achieved by changing the structure of intestinal microbiota (16). Since it can be digested more effectively in the gastrointestinal tract, COS can provide better bacterial regulation effects than chitin or chitosan (17). It stimulated the growth of Lactobacillus, and induced significantly decrease of genera Lachnospiraceae NK4A136, Alistipes, Helicobacter, Ruminococcus and Odoribacter, while Lachnospiraceae UCG 001 and Akkermansia increased under this circumstances (18). When combined with resistant starch, COS administration can reduce protein fermentation markers, such as H_2_S, ammonia, phenol and indole, as well as increasing the excretion of bile acids in feces, the thickness of the mucosal layer, and the production of SCFA (19). It is reported that COS also closely relates to the occurrence of diabetes in some studies (20), which fully exhibits the benefits of COS intake. In addition, COS also represses the growth of *E. coli*, alters the abundance of phyla Bacteroidetes, Verrucomicrobia, Proteobacteria and Firmicutes, and affects Bacteroides–Prevotella and Enterobacteriaceae in the same way (19).

However, despite its wide application, the possible side effect of COS needs to be further evaluated, and the effect of COS on the intestinal microbiota and its roles in improving metabolic syndrome are still need to be confirmed. In this study, we first verified the prebiotic effect of COS in controlling body weight and blood glucose, then analyzed the influence of COS on the intestinal microbiota, and the results revealed that the effects of COS were closely related to some bacteria in the microbiota. This study provides new evidence for the prebiotic effect of COS.

## Materials and Methods

### Ethical Approval

This study was approved by the Medical Ethic Committee of School and Hospital of Stomatology, Shandong University (Approval ID: GR20180501). According to the institutional guidelines for animal research, all animals are carefully maintained before and during the experiment. All animal experiments were carried out in compliance with the U.K. Animals (Scientific Procedures) Act 1986 and the associated guidelines (EU Directive 2010/63/EU) for animal experiments.

### Animal Feeding and Handling

Six-week-old C57BL/6J male mice (*n* = 40) were purchased from the Laboratory Animal Center of Shandong University. The mice are normal laboratory mice, without gene function abnormalities and diseases, and no antibiotics have been used. All mice were fed individually at a constant temperature (23 ± 2°C) and a 12-h light/12-h dark cycle. After being fed with a normal chow diet for 2 weeks to allow for adaption to the environment, all mice were randomly divided into four groups and treated for another 56 days: low-fat diet group (group L, fed with 4.3% fat, 5% cellulose, w/w); low-fat diet + COS group (LC group, fed with 4.3% fat, 5% COS, w/w) (21–23); high-fat diet group (group H, fed with 35.4% fat, 5% cellulose, w/w); and high Fat diet + COS group (HC group, fed with 35.4% fat, 5% COS, w/w) (Energy contained in foods was shown in Supplementary Table 1). Both low-fat and high-fat mouse feeds were purchased from Research Diets Inc., New Brunswick, NJ, USA. Their product numbers were D12450J and D12492, respectively. Chitosan oligosaccharides (COS) was purchased from Bozhihuili Biotechnology Co., Ltd., Qingdao, Shandong, China, the product number was 2018100801CWF, and the molecular weight was <1500Da.

### Testing the Amount of Obesity-Related Factors

Throughout the experiment, the weight and food intake of the mice were monitored every 3 or 4 days. The fasting blood glucose was measured from the tail vein by an ACCU-CHEK Performa glucose analyser (Roche, Switzerland) after the mice had fasted for 6 h. The oral glucose tolerance test (OGTT) was performed at the beginning and end of the experiment, the method is to measure the amount of mice blood glucose at 0, 15, 30, 60, 90, and 120 min after gavage with 150 μl glucose solution (0.5 g/ml). The Homeostasis model assessment-insulin resistance (HOMA-IR) was employed to calculate the level of insulin resistance. The calculation formula is fasting serum glucose (mmol/L) × fasting serum insulin (mIU/L)/22.5. The levels of fasting serum insulin and leptin were tested by the Mouse leptin (insulin) kits (Bio-Swamp, China). Serum total cholesterol, triglycerides, low-density lipoprotein cholesterol and high-density lipoprotein cholesterol were determined by the corresponding kits (Jiancheng, China). The amount of short-chain fatty acids in the cecum of mice was tested by the Elisa kit (Jianglai, China).

### Histological Analysis

The epididymal white adipose tissue (Epi-WAT) and liver samples were fixed in a 4% paraformaldehyde solution in phosphate buffered saline and embedded in paraffin. Tissue sections (5 μm) were then stained with haematoxylin and eosin (HE) and visualized under a microscope (Olympus BX51, Japan). The sizes of the adipocytes were measured by ImageJ (National Institutes of Health, USA).

### Quantitative Real-Time PCR

The tissue was first cut into small pieces with scissors and then ground with a grinder (JXFSTPRP series automatic sample grinding machine, Jingxin company, Shanghai, China) according to the instruction manual. Total RNA was extracted with TRIzol® (CWBIO, China) according to the manufacturer's instructions. The TRIzol® reagent can extract RNA from tissues and cells efficiently, and contains a protocol for purification of sample RNA from human, animal, plant or bacterial sources. The TRIzol® reagent maintains RNA integrity by inhibiting RNase activity during sample homogenization, while destroying cells and dissolving cellular components. The total RNA separated by TRIzol® reagent does not contain protein and DNA contamination. The mRNA concentration was determined using an ultramicrospectrophotometer (Thermo, Waltham, MA, USA). Equal amounts of mRNA from each mouse were pooled to normalize individual differences, and mRNA was reverse-transcribed to cDNA using a HiFiScriptcDNA Synthesis Kit (CWBIO). Quantitative real-time PCR was performed with UltraSYBR Mixture (CWBIO) on a Light Cycler 96 Real-Time PCR System (Roche, Switzerland), and amplified for 45 cycles of denaturation at 95°C for 10 s, annealing at 60°C for 30 s and extension at 72°C for 32 s. The housekeeping gene GAPDH is used as an internal reference for gene expression, and the primers used for amplification were shown in Supplementary Table 2.

### 16S rRNA Sequencing and Data Analysis

The fecal samples were collected from the four groups of mice on days 0, 7, 28, and 56, and cecal content samples were collected from each mouse when the mice were sacrificed. Total genomic DNA in the sample is extracted by the CTAB/SDS method. The DNA concentration and purity were monitored on a 1% agarose gel. Based on the concentration, DNA was diluted to 1 ng/μL using sterile water. All PCRs were performed with a 30 μL reaction volume containing 15 μL Phusion® high-fidelity PCR master mix (New England Biolabs), 0.2 μM forward and reverse primers, and approximately 10 ng template DNA. Thermal cycling included initial denaturation at 98°C for 1 min, 30 cycles of denaturation at 98°C, annealing at 50°C for 30 s, and extension at 72°C for 30 s. The final stage was 72°C for 5 min. The same volume of 1 loading buffer (containing SYB green) was mixed with the PCR products and electrophoresed on a 2% agarose gel for detection. Then, the mixed PCR products were purified using GeneJETTM Gel Extraction Kit (Thermo Scientific). The V3-V4 region of 16S rRNA was sequenced on the Ion S5 sequencing platform (Novogen Co., Ltd., Beijing) according to standard operating procedures. The average sequencing depth of each sample was 66,052 reads. Taxonomy was assigned with RDP (Ribosomal Database Project), and OTUs were generated with UPARSE, which sorted 12,087,520 quality-filtered sequences into 2203 OTUs at 97% sequence homology. Bioinformatical processing was performed with Qiime v2 and Usearch. The analysis in this study adopted the methods of relative abundance to normalize the compositional and sparse data, and Permutation Test was used to calculate differential abundance among groups. Spearman correlation analysis was employed to analyze the relationship between related indicators. In all groups, the bacterial taxa with the average relative abundance≥0.02% and the frequency≥50% were selected for Spearman correlation analysis. The species and metabolic syndrome-related indices with *p* ≤ 0.01 were regard as significant correlation and heatmaps and network graphs were used for exhibition.

### Statistical Analysis

All data are expressed as the mean ± standard deviation (SD) unless otherwise specified. Statistical tests were performed by GraphPad Prism software (version 6, MacKiev Software, Boston, MA, USA) and R software (version 3.5.1). Differences between two groups were checked by Permutation Test using the R package “Deducer,” while statistics among more than two groups were analyzed by one-way ANOVA with Tukey's Honestly Significant Difference (Tukey's HSD) test using GraphPad. *P* < 0.05 was considered statistically significant.

## Results

### COS Improves Fat Accumulation and Dyslipidemia Without Negative Effects

**Figure 1:**
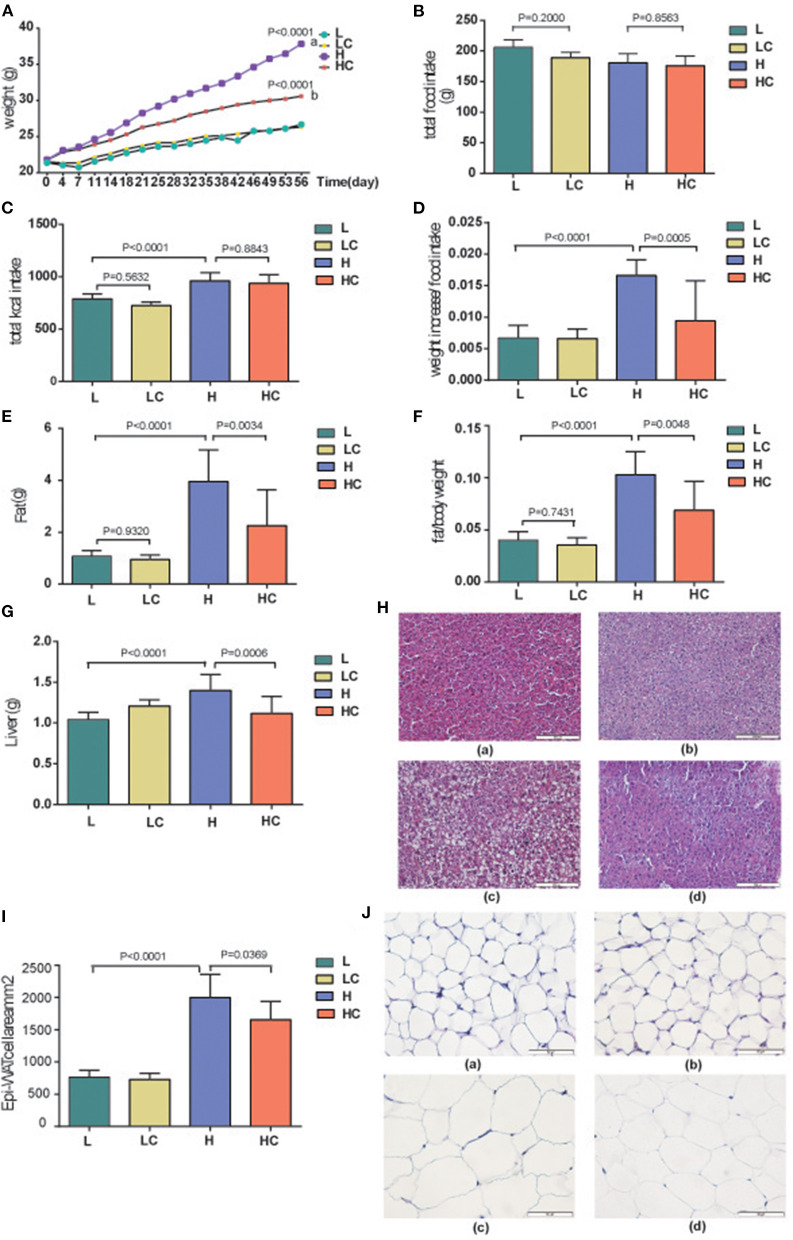
Effects of COS on Body weight and liver fat. **(A)** Changes in mouse body weight over time; **(B)** Food intake of mice in each group; **(C)** Calculated energy intake of each group; **(D)** Weight increase per unit different diet; **(E)** Average fat weight of each group; **(F)** Average BFR, body fat rate; **(G)** Average liver weight of different groups; **(H)** H&E staining of liver in the L (a), LC (b), H (c), and HC (d) groups; **(I)** The size of Epi-WAT cells; **(J)** H&E staining of Epi-WAT in L (a), LC (b), H (c), and HC (d) groups. Groups: Group L (low-fat diet group, fed with 4.3% fat, 5% cellulose); LC group (low-fat diet + COS group, fed with 4.3% fat, 5% COS); group H (high-fat diet group, fed with 35.4% fat, 5% cellulose); and HC group (high Fat diet + COS group, fed with 35.4% fat, 5% COS).

To determine the effect and safety of COS under the circumstance of high-fat and low-fat diets, the body weights and food intakes of mice in each group were monitored from 0 to 56 days. The results showed the average weight gain of the HC group was significantly slower than H group (Figure 1A), yet the food intake exhibited no divergence among the four groups (Figure 1B). We then calculated the energy intake, which showed similarity between the H and HC groups and higher than L and LC groups (Figure 1C). COS markedly reduced the weight gain per unit of dietary intake in high-fat diet groups, while no distinction was observed in the low diet groups (Figure 1D). The body fat rate evaluation showed that the fat weight and rate of the low-fat diet group were greatly lower than those of the high-fat diet group, while the HC group was clearly lower than that of the H group in the high-fat diet groups (Figures 1E,F). Additionally, the average liver weight of the H group was found higher than HC groups and L group (Figure 1G). The H&E staining on liver tissue even showed that the fat accumulation in the liver of HC group was similar to those of the low-fat diet groups (Figure 1H). Then, the epididymal white adipose tissue (Epi-WAT) was examined by H&E staining. The results indicated that the Epi-WAT cell area of the low-fat feeding groups was significantly lower than that of the high-fat feeding groups, and COS supplementation significantly reduced the area of Epi-WAT cell in the high-fat diet group (Figure 1I). The size of fat cells also indicated constitutive variation of H to HC and L groups (Figure 1J). These results indicate that COS supplementation could improve fat cell hypertrophy caused by a high-fat diet.

**Figure 2:**
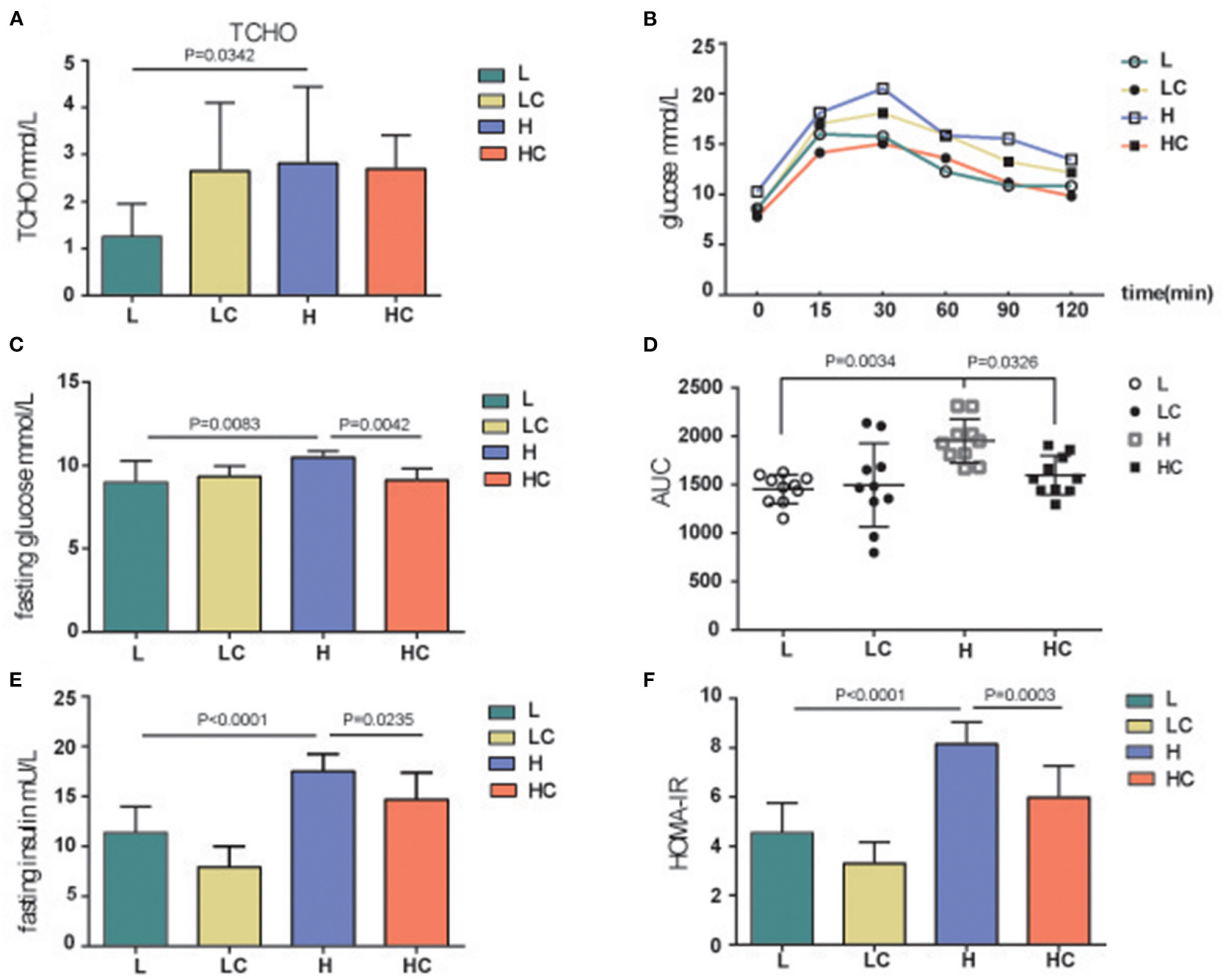
Blood glucose, insulin and physiological metabolites analysis. TCHO **(A)**, Glucose **(B)**, Fasting glucose **(C,D)**, Fasting insulin **(E)** and insulin resistance (**F**, HOMA-IR) levels of different groups. Groups: Group L (low-fat diet group, fed with 4.3% fat, 5% cellulose); LC group (low-fat diet + COS group, fed with 4.3% fat, 5% COS); group H (high-fat diet group, fed with 35.4% fat, 5% cellulose); and HC group (high Fat diet + COS group, fed with 35.4% fat, 5% COS).

When the mice were given a high-fat diet for a period of time, metabolic dyslipidemia might be induced; the amount of blood total cholesterol (TCHO) demonstrated a variation between the L and H groups (Figure 2A). However, the shift in some blood lipid metabolites was not significant, such as low and high density lipoprotein cholesterol (LDL-C and HDL-C), although their average values exhibited fluctuations (Supplementary Figures 1A–D). Meanwhile, the addition of COS did alleviated dyslipidemia; the level of TCHO increased, while the levels of HDL-C increased accordingly (Supplementary Figures 1A–D). These results indicate that COS can restore certain fat parameters disorders induced by high-fat diet. Next, the blood glucose deviation instigated by high-fat diet was restored to normal levels by COS (Figures 2B,C), whereas the concentrations of serum triacylglycerols (TGs) and leptin showed no variation among the four groups (Supplementary Figures 1A,D). The fasting blood glucose and insulin levels were further tested. The blood glucose of the H group was found to be significantly higher than that of the L group, while its level in the HC group was restored to be comparable to that of L group (Figure 2D). Similarly, the fasting insulin level of H group was also significantly divergent from that of COS-treated HC group and low-fat fed L group (Figure 2E); the HOMA-IR results indicated insulin resistance level was also significantly higher in the H group than in the L group and the HC group (Figure 2F). It is worth noting that the above-mentioned items showed no variation between the L and LC groups, indicating that COS may not affect the energy absorption and metabolism of mice in the case of low-fat diet.

### The Effects of COS on Gut Microbiota

**Figure 3:**
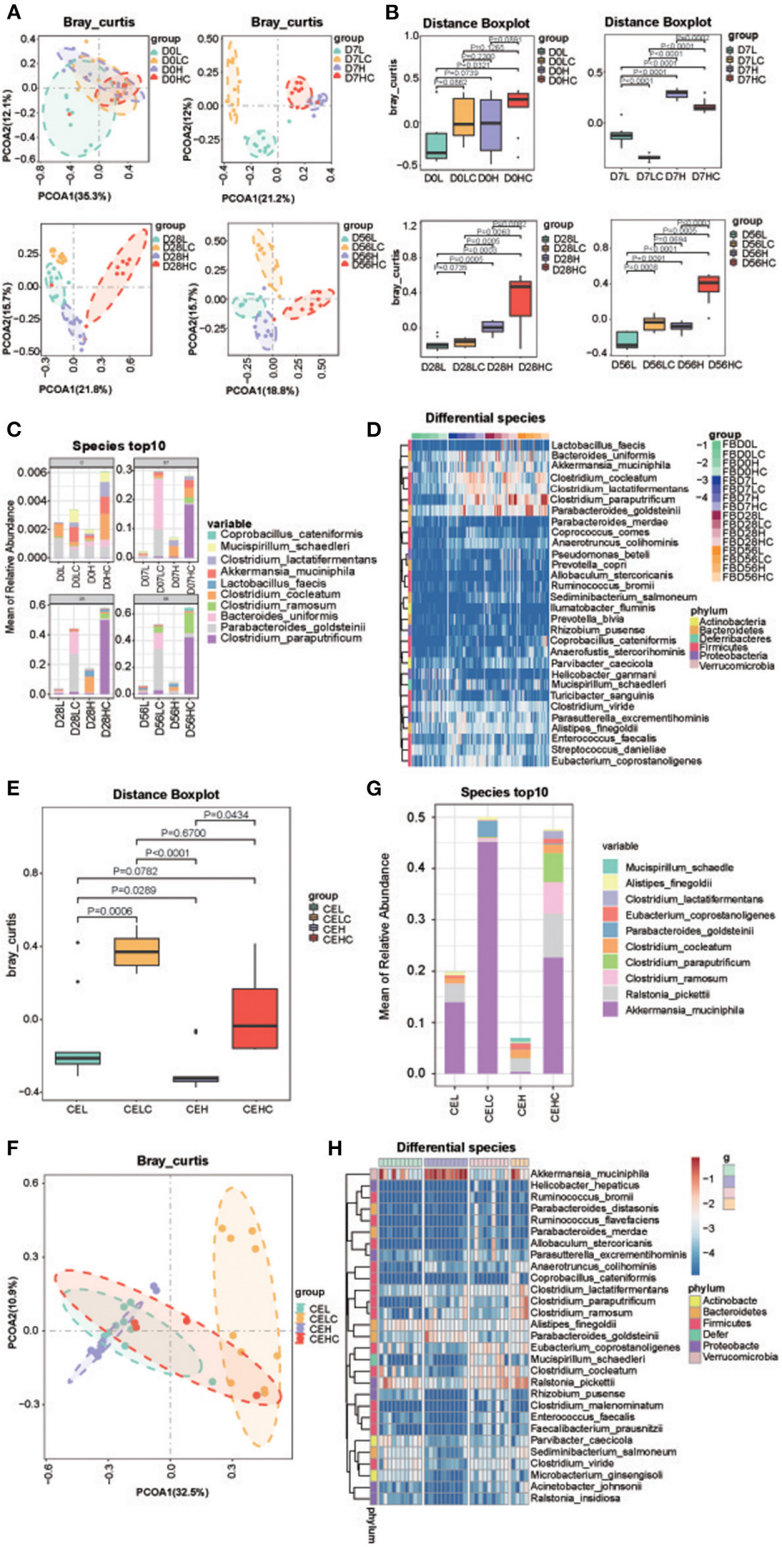
Structure of the gut microbiome. **(A)** PCOA analysis of the fecal microbiota; **(B)** Bray Curtis distance of the fecal microbiota; **(C)** Top 10 dominant fecal microbes over time in each group; **(D)** Heatmap of differential fecal species at the same time point; **(E)** PCOA analysis of the cecum microbiota; **(F)** Bray Curtis distance of the cecum microbiota; **(G)** Top 10 dominant fecal microbes over time in each group; **(H)** Heatmap of differential cecum microbes at the same time point. Groups: Group L (low-fat diet group, fed with 4.3% fat, 5% cellulose); LC group (low-fat diet + COS group, fed with 4.3% fat, 5% COS); group H (high-fat diet group, fed with 35.4% fat, 5% cellulose); and HC group (high Fat diet + COS group, fed with 35.4% fat, 5% COS).

To explore the role of intestinal microbiota in COS-induced recovery of obesity and metabolite deviation, 16S rRNA analysis was performed toward the fecal samples obtained at day 0, 7, 28 and 56; and the caecal microbiota of day 56 was also tested. Supplementary Figure 2 showed the changes of α-diversity indexes after COS treatment. The rarefaction curve indicated that all samples tended to be saturated, suggesting the OTUs covered most of the bacterial species in the mouse intestine. The Shannon indices showed that the α-diversities of the groups with COS were lower than the corresponding control groups, and the *p*-value decreased gradually over time, implying that COS may induce reduction of within-sample microbial diversity (Supplementary Figure 3A). The Principal coordinate analysis (PCoA) results exhibited that all dots of COS fed groups LC and HC got more separated from their corresponding control groups L and H as time increased (Figure 3A). The distance boxplot of Bray Curtis exhibited the enhancement of between-sample microbial diversity of intestinal microbiota concomitant with the consumption of COS (Figure 3B). The Adonis/Permanova metric was used to analyze the significance of PCOA1 differences between groups. The *p*-values were 0.001, 0.001 and 0.001 on the 7, 28, and 56 days, respectively.

Then the compositions of fecal bacteria were interrogated, the result revealed the number of phylum in mice intestine remained unchanged after COS treatment; and this phenomenon was observed in all 7, 28, and 56 d groups. At the genus level, the number of genera in the fecal microbiota decreased after COS treatment, LC and HC decreased by 52 and 32 genera, respectively, compared with their controls; but at 7 and 28d time points, the HC groups contained more genera than the H groups. From a structural point of view, the dominant genera in feces transitioned from Lactobacillus to Lactobacillus, Clostridium_XVIII and Bacteroides with the extension of high-fat feeding time (Supplementary Figure 3B). Compared with the H group at the same time, Clostridium_sensu_stricto in the HC group increased, while Lactobacillus and Bacteroides decreased. Similarly, COS also induced less Lactobacillus in the low-fat diet groups, but genus Bacteroides increased together with Parabacteroides in LC group. The changes in abundance at the level of genus were also verified in the Heatmap of each genus (Supplementary Figure 3C). Similar to genus level, the decrease in the number of species in mice feces occurred in the 56th day group; but in the 7th and 28th day groups, the HC group contained more species than the H group. Generally, COS induced more *Clostridium paraputrificum* and *Clostridium ramosum* in the high-fat diet groups, while the content of *Clostridium cocleatum* decreased (Figure 3C). In the low-fat diet groups, *Parabacteroides goldsteinii* and *Bacteroides uniformis* enhanced their abundance in LC group. The changes in abundance at the level of species were also verified in the Heatmap of each species (Figure 3D and Supplementary Figure 3D). It is worth noting that the abundance of *Akkermansia muciniphila* is induced by COS in both high-fat and low-fat diets conditions, implying the probiotic effect of COS.

As a comparison, the effect of COS on the cecum microbiome was also analyzed and its differential bacteria were investigated. The Shannon index analysis showed the α-diversity of ceacal microbiota decreased in the COS fed groups (Supplementary Figure 4A). The Distance boxplot of Bray Curtis indicated that the distributions of the COS fed groups were significantly divergent from their control groups (Figures 3E,F). At the predicted species level, COS feeding enriched *Akkermansia_muciniphila, Clostridium paraputrificum* and *Clostridium ramosum*, while the content of *Clostridium cocleatum* and *Ralstonia_pickettii* decreased in the high-fat diet groups, which is consistent with the results of fecal microbiota (Figure 3G). In the low-fat diet groups, *Parabacteroides goldsteinii* and *Akkermansia_muciniphila* increased their abundance concomitant with COS additive, while *Clostridium cocleatum* and *Ralstonia_pickettii* reduced their contents. The changes in abundance of species were verified in the Heatmap of each species (Figure 3H). The results at the species level are consistent with those at the genus level (Supplementary Figures 4B,C). Noticeably, the COS-induced bacterial changes in the cecum were fantastically similar to that of feces whether in the high- and low-fat diet groups, deepening our understanding of the prebiotic effects of COS.

### The Interaction of Bacteria

**Figure 4:**
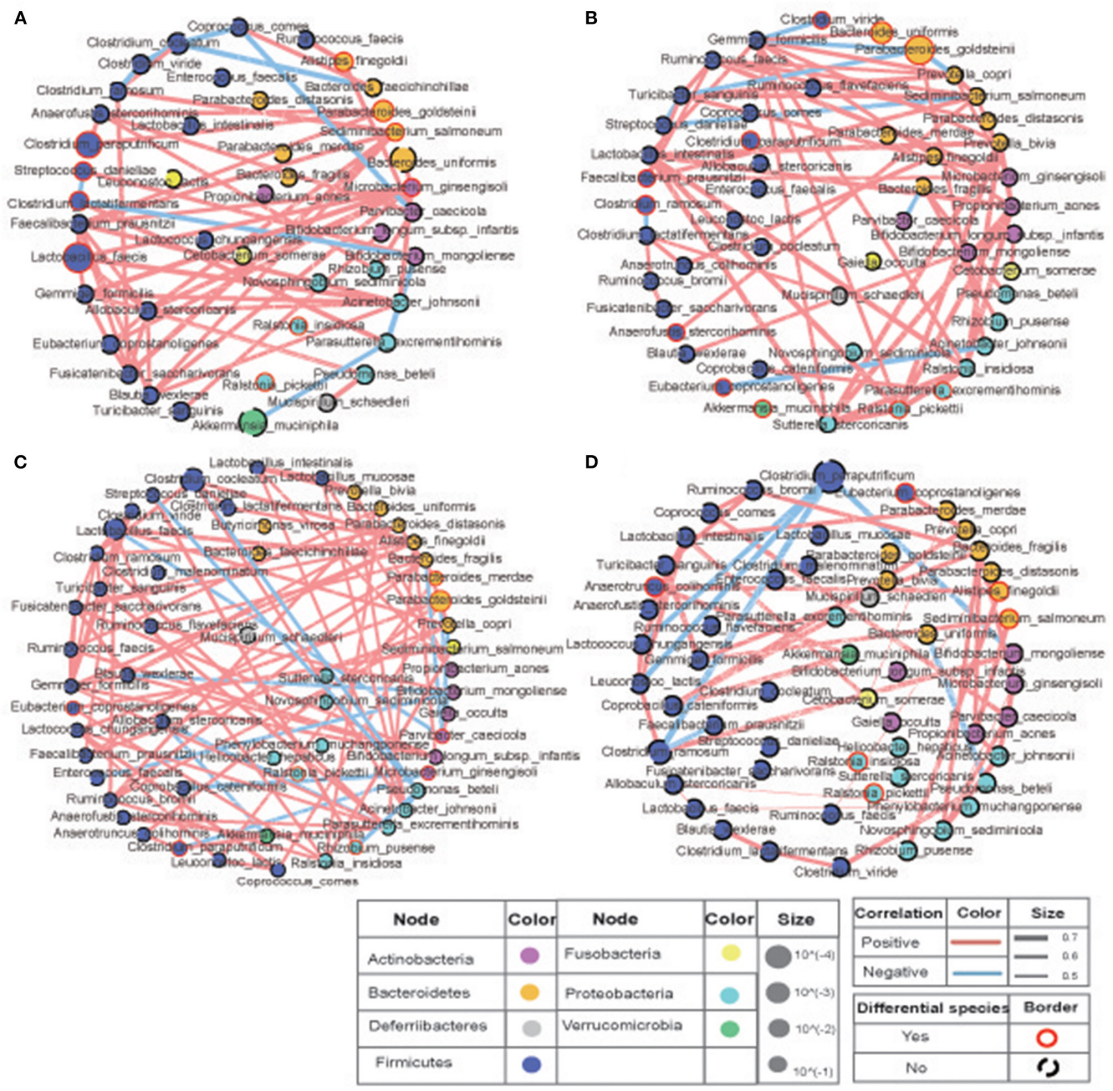
Correlations of the bacteria in murine intestine. The size of the circle represents the relative abundance, the color of the circle represents the phylum to which it belongs, the red line is positive correlation, the blue line is negative correlation. Compared with the low-fat diet **(A)** L and **(B)** LC groups, the high-fat diet **(C)** H and **(D)** HC groups had more complex interactions between the bacteria and tremendous change occurred in their relationship. COS stimulation enabled *Clostridium ramosume* to establish positive interactions with *Faecalibacterim prausnitzii* and *Clostridium paraputrificum*; *Clostridium paraputrificum* also established positive interactions with *Lactococcus chungangensis* and *Bifidobacterium mongoliense* **(B,D)**. Groups: Group L (low-fat diet group, fed with 4.3% fat, 5% cellulose); LC group (low-fat diet + COS group, fed with 4.3% fat, 5% COS); group H (high-fat diet group, fed with 35.4% fat, 5% cellulose); and HC group (high Fat diet + COS group, fed with 35.4% fat, 5% COS).

To study the correlation between the bacteria in the two sites with or without COS stimulation, |Spearman correlation|≥0.7 and *q* ≤ 0.01 were set as the filter parameters, and the species with average relative abundance ≥0.02% were selected. The results showed that close correlations existed among the fecal bacteria, and clear variations in the pattern of bacterial correlations were observed in the COS feeding groups (Figure 4). Compared with the low-fat diet L group, the high-fat diet H group had more complex interactions between the bacteria and tremendous change occurred in their relationship. Specific for those bacteria associated with *Akkermansia muciniphila, Clostridium ramosume, Clostridium cocleatum, Parabacteroides goldsteinii* and *Clostridium paraputrificum*, only the relationships of *Clostridium ramosume* and *Fusicatenibacter saccharivorans*, as well as *Parabacteroides goldsteinii* and *Anaeofustis stercorihominis* were shared in both groups (Figure 4). Similarly, COS feeding also greatly changed bacterial interactions in the high-fat diet group, reducing the relationship in the microbiota, such as shearing bacteria interactions with *Clostridium cocleatum* and *Parabacteroides goldsteinii*. It is also worth noting that COS stimulation enabled *Clostridium ramosume* to establish positive interactions with *Faecalibacterim prausnitzii* and *Clostridium paraputrificum*; *Clostridium paraputrificum* also established positive interactions with *Lactococcus chungangensis* and *Bifidobacterium mongoliense* (Figures 4B,D). Since *Faecalibacterim prausnitzii, Akkermansia muciniphila, Parabacteroides goldsteinii, Lactococcus spp.*, and *Lactococcus spp*. have been reported in the previous literature to have beneficial effects on weight loss, thereby these results suggest that COS may improve metabolic syndrome and physiological parameters through intestinal bacteria.

### The Effect of COS on Intestinal Cells

**Figure 5:**
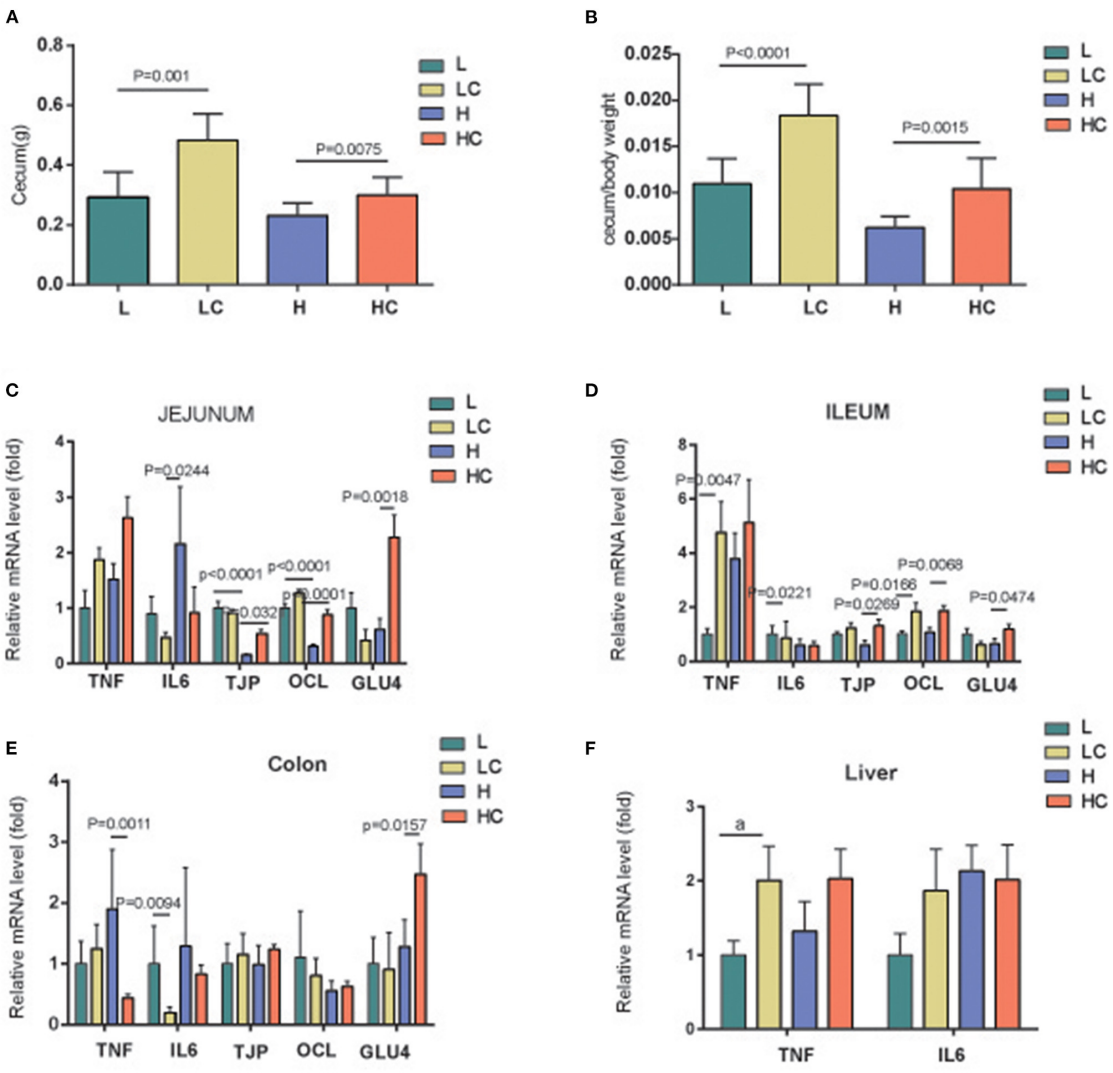
Gene expression of the cells in gut and liver. Cecum weight **(A)**, cecum/body weight **(B)** of each group, and gene expressions in jejunum **(C)**, ileum **(D)**, colon **(E)** and liver **(F)**. Groups: Group L (low-fat diet group, fed with 4.3% fat, 5% cellulose); LC group (low-fat diet + COS group, fed with 4.3% fat, 5% COS); group H (high-fat diet group, fed with 35.4% fat, 5% cellulose); and HC group (high Fat diet + COS group, fed with 35.4% fat, 5% COS).

To study the mechanism of the effect of COS on intestinal microbiota and physiological status, the weight of the cecum of each group was recorded and compared between the groups. The weight of the cecum reflects the growth and development of animals and the physiological and ecological conditions, and can be used as an approximate index for animals to adapt to the environment (24, 25). The results revealed that the cecum weight and the cecum/body weight ratio of the COS-addition groups increased significantly in comparison to their control feeding groups (Figures 5A,B). To explore the variations of other environmental factors, gene expression of inflammatory factors TNFα and IL-6, tight junction proteins TJAP1 and OCL, as well as facilitated glucose transporter GLUT4 in jejunum, ileum and colon were also monitored. Compared with the H group, the expression of gene *tjp* and *ocl* in the jejunum and ileum of the HC group were significantly increased, while the expression variation of *glut4* in these two parts was inconsistent (Figures 5C,D); COS feeding decreased the level of TNFα in the colon, but the level of GLUT4 in this site increased (Figure 5E). The expression of *il6* in colon in the LC group was significantly lowered than the L group, suggesting that COS may have an anti-inflammatory effect in this site (Figure 5E); but it did not affect the expression of TNFα and IL-6 or had opposite effects in the other two parts (Figures 5C–E). In the liver, there was no difference in the expression of TNF and IL-6 among the four groups (Figure 5F). The above results indicated that COS supplements did not stimulate inflammation, but could improve the expression of genes related to the intestinal barrier and glucose transport, which could be the trigger and consequence of the shiftiness in gut microbiota induced by COS.

### Associations of Intestinal Bacteria With Metabolic Syndrome-Related Indices

**Figure 6:**
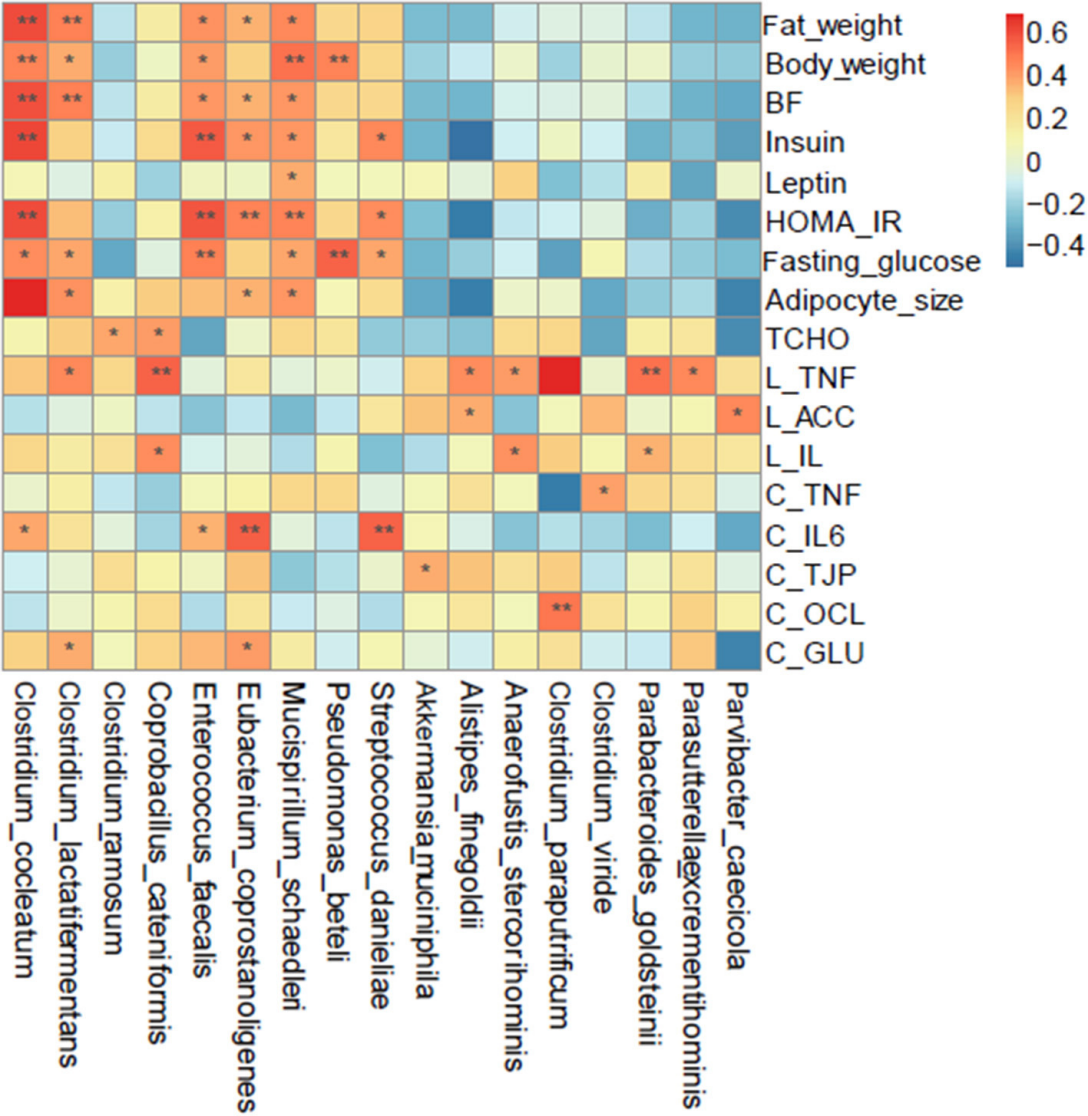
Spearman correlation analysis of the bacteria at the species level and metabolic syndrome-related indices. Groups: Group L (low-fat diet group, fed with 4.3% fat, 5% cellulose); LC group (low-fat diet + COS group, fed with 4.3% fat, 5% COS); group H (high-fat diet group, fed with 35.4% fat, 5% cellulose); and HC group (high Fat diet + COS group, fed with 35.4% fat, 5% COS).

To explore the possible relationship between COS, microbiota and clinical parameters, Spearman correlation with the parameter of *p* ≤ 0.05 and occurrence rate ≥30% were employed to analyze the prevalent taxa (≥0.02%) at the genus and species levels. The results showed that a variety of microbes altered in response to COS were closely correlated to clinical indexes; *Akkermansia muciniphila, Parabacteroides goldsteinii* and *Clostridium paraputrificum* were positively related to cecum weight and proportion (Figure 6). Moreover, *Clostridium cocleatum* and *Clostridium ramosume* were differentially correlated with the obesity indexes; *Clostridium cocleatum* was closely associated with body and fat weights, adipocyte size, BF, HOMA-IR, glucose and insulin (Figure 6). *Clostridium ramosume* was not related to the above parameters, but was correlated with Tcho, CBF and cecum weight. *Akkermansia muciniphila, Parabacteroides goldsteinii, Clostridium ramosume* and *Clostridium cocleatum* shift in different directions under the induction of COS, this may be the reason why COS ameliorates metabolic dyslipidemia and adipocyte hypertrophy. Succeedingly, the relationship between the intestinal bacteria and gene expression of inflammatory factors TNFα and IL-6, tight junction proteins TJAP1 and OCL, as well as facilitated glucose transporter GLUT4 in jejunum, ileum and colon were explored by Spearman analysis. *Akkermansia muciniphila* and *Clostridium paraputrificum* induced by COS were positively related to the expression of tight junction proteins TJP and OCL; while COS reduced species, such as *Clostridium cocleatum, Clostridium lactatifermentans, Eubacterium coprostanoligenes, Clostridum viride*, and *Streptococcus danieliae*, were positively related to inflammation related genes TNF and IL-6 (Figure 6). These results suggest that COS improves physiological parameters through intestinal bacteria, further illustrating the prebiotic effects of COS.

## Discussion

The intestinal microbiota has a profound influence on metabolism, tissue development, and homeostasis of the intestinal immune system (26). Various mechanisms between gut microbes and immune response have been proposed. For example, changes in the gut microbiome may promote intestinal permeability and alter the production of butyric acid and lipopolysaccharide (LPS), while the levels of butyric acid and LPS may regulate immune response and inflammation (26). In addition, changes in intestinal microbiota may change energy homeostasis, promote fat accumulation, and regulate the host's inflammatory state (27). Obesity has become a prevalent social problem and possesses a variety of adverse effects on human health (28). In this study, we induced obesity by feeding mice with high fat, and measured their clinical parameters to verify the prebiotic effect of COS on reducing obesity. The results showed that COS reduced body and liver fat accumulation caused by high-fat diet. Moreover, mice supplemented with COS ameliorated metabolic dyslipidemia and adipocyte hypertrophy. Moreover, COS feeding may also improve the expression of genes related to the intestinal barrier and glucose transport, but does not stimulate inflammation. Next, the effect of COS on the intestinal microbiota was examined. In the high-fat diet group, COS induced more *Clostridium paraputrificum* and *Clostridium ramosum*, while the content of *Clostridium cocleatum* decreased. In the low-fat diet group, *Parabacteroides goldsteinii* and *Bacteroides uniformis* in the LC group increased their abundance. Noticeably, COS can induce a large amount of *Akkermansia muciniphila* whether under high-fat or low-fat diet conditions, confirming the probiotic effect of COS. Additionally, COS feeding also enabled *Clostridium ramosume* to establish positive interactions with *Faecalibacterim prausnitzii* and *Clostridium paraputrificum*; *Clostridium paraputrificum* established positive interactions with *Lactococcus chungangensis* and *Bifidobacterium mongoliense*, thereby these results suggest that COS improves metabolic and physiological parameters probably through the microbiota.

COS has exhibited a beneficial effect on the body in previous studies, and this effect have been verified in our research. For example, COS inhibited the activity of pancreatic lipase and reduced the absorption of intestinal fat in combination with bile acids, as well as increased the excretion of fecal fat (29). COS also down-regulated apolipoprotein B and ghrelin in the stomach and inhibit adipocyte differentiation by up-regulating adiponectin (30). Although some other indices were tested, the effect of COS in inhibiting fat accumulation has been verified in this research. Mechanically, COS can activate AMPK in muscle cells and other fat cells (23), inhibit hepatic gluconeogenesis and stimulate glycogen synthesis in the liver by inhibiting the expression of p38 MAPK and phosphoenolpyruvate carboxykinase (PEPCK), as well as activate AMPK and up-regulate glucokinase expression (31). In addition, COS demonstrated an effect to inflammation and related damage in the IBD by inhibiting NF-κB-mediated inflammation and apoptosis of intestinal epithelial cells. Our research revealed that COS feeding could also improve the expression of genes related to the intestinal barrier and glucose transport, and found that COS did not stimulate inflammation. The specific signaling pathways it affects will be explored in the future. In terms of microbial modulation, studies have proven that COS stimulated the growth of *Lactobacillus rhamnosus* (17), and induced significantly decrease of genera Lachnospiraceae NK4A136, Alistipes, Helicobacter, Ruminococcus and Odoribacter, while Lachnospiraceae UCG 001 and Akkermansia increased under this circumstances (18). COS also inhibits the growth of *E. coli* (17), alters the abundance of phyla Bacteroidetes, Verrucomicrobia, Proteobacteria and Firmicutes, and affects Bacteroides–Prevotella and Enterobacteriaceae in the same way (19). However, the results in our study are significantly different from previous results in terms of the bacterial species and bacterial interaction induced by COS. Our data indicated that COS improved the levels of *Parabacteroides goldsteinii, Coprobacillus cateniformis* and *Akkermansia muciniphila* in mice while decreasing overnutrition-related microbes such as *Clostridium cocleatum, Clostridium lactatifermentans, Eubacterium coprostanoligenes, Streptococcus danieliae* and *Clostrdium viride*. Besides that *Akkermansia muciniphila* had many effects, such as metabolic and immune disease improvement and cancer therapy (32), *Parabacteroides goldsteinii* was also a probiotic that could improve glucose metabolism, reduce intestinal inflammation and obesity (33–35). Furthermore, *Akkermansia muciniphila, Parabacteroides goldsteinii* and *Clostridium paraputrificum* were proved to be positively related to cecum weight and proportion in this study; *Parabacteroides goldsteinii* was also found positively related to SCFAs. Moreover, *Clostridium cocleatum* and *Clostridium ramosume* were differentially correlated with the obesity indexes; *Clostridium cocleatum* was closely associated with body and fat weights, adipocyte size, BF, HOMA-IR, glucose and insulin, while *Clostridium ramosume* was correlated with Tcho, CBF and cecum weight. These results imply that COS ameliorate metabolic syndrome at least partially through the microbiota, further probiotic feeding and receptor knockout experiments are needed to verify these results in the future.

Controlling and reducing adverse drug reactions is a key issue to monitor the possible bad effects of drug. The reliability, safety and purity evaluations of a drugs or biomedical equipment are indispensable tasks. COS was proved to have no mutagenic potential (36), and systemic toxicity analysis indicated no mortality and allergenicity at the average lethal dose (LD50) of >10 g/kg (37). Research on colorectal cancer revealed that it had no adverse effect on renal and hepatic functions in animal models (38). In order to obtain clinically approved for COS, accumulation of data in animal models is needed. In this study, we found that COS feeding did not reduce the weight of mice on a low-fat diet, nor did they reduce their food and energy intake in addition to improving the metabolism of mice on a high-fat diet. Moreover, hepatocyte morphologies in the mice of HC and LC group were all similar to those of L group; COS supplementation could improve fat cell hypertrophy caused by a high-fat diet, but there is no difference between the two groups fed on a low-fat diet. In addition, COS did not stimulate inflammation in mice, but improved their function related to intestinal barrier and glucose transport. Finally, no novel pathogenic bacterium was observed in the intestines of mice fed with COS no matter in high-fat or low-fat diets conditions, proving that it can be used with confidence. In summary, our research results provide new evidence for the prebiotic effects of COS.

## Data Availability Statement

The datasets presented in this study can be found in online repositories. The names of the repository/repositories and accession number(s) can be found at: NCBI SRA; PRJNA656407.

## Ethics Statement

The animal study was reviewed and approved by The Medical Ethic Committee of School and Hospital of Stomatology, Shandong University.

## Author Contributions

QF designed and supervised this study. SL, YW, DT, and RD performed experiments. YW analyzed the data and plotted figures. SL and QF wrote and edited the manuscript. All authors contributed to the article and approved the submitted version.

## Funding

This study was supported by the National Natural Science Foundation of China (No. 82071122), The Construction Engineering Special Fund of Taishan Scholars of Shandong Province (tsqn201909180), and National Key R&D Program of China (No. 2017YFB0405400) and the Program of Excellent young scholars of Shandong University.

## Conflict of Interest

The authors declare that the research was conducted in the absence of any commercial or financial relationships that could be construed as a potential conflict of interest.

## Publisher's Note

All claims expressed in this article are solely those of the authors and do not necessarily represent those of their affiliated organizations, or those of the publisher, the editors and the reviewers. Any product that may be evaluated in this article, or claim that may be made by its manufacturer, is not guaranteed or endorsed by the publisher.
