# Supplementary material for: Chitosan Oligosaccharide Ameliorates Metabolic Syndrome Induced by Overnutrition via Altering Intestinal Microbiota
Source: Front Nutr. 2021 Oct 1;8:743492. doi: 10.3389/fnut.2021.743492 (PMC8517441; doi:10.3389/fnut.2021.743492)
Supplement: Supplementary file 1 [file Data_Sheet_1.DOCX]

Supplementary material

Supplementary Figure 1


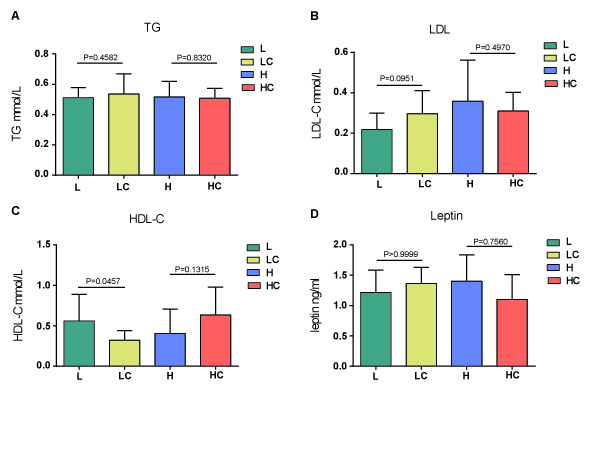


Supplementary Figure 1. Biochemical monitoring of metabolic syndrome. A. Average serum triglyceride (TG) levels of each group. B. Average Low density lipoprotein cholesterol (LDL-C) levels of each group. C. Average serum High density lipoprotein cholesterol (HDL-C) levels of each group. D. Average serum leptin levels of each group.

Supplementary Figure 2


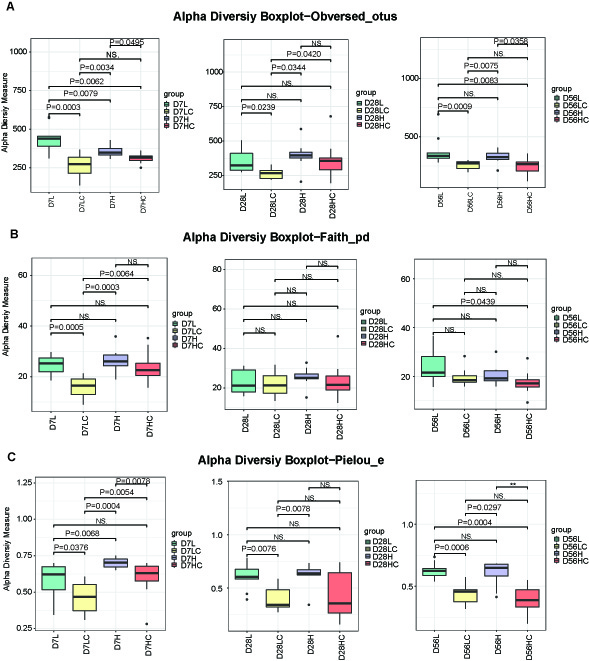


Supplementary Figure 2. α-diversity exhibited by box plot: the α-diversity index includes: A. observed otus: the types of OTUs; B. Faith_pd: phylogenetic diversity, taking into consideration of the distribution of samples on the evolutionary tree, reflecting species richness; C. Pielou_e: species uniformity index.

Supplementary Figure 3


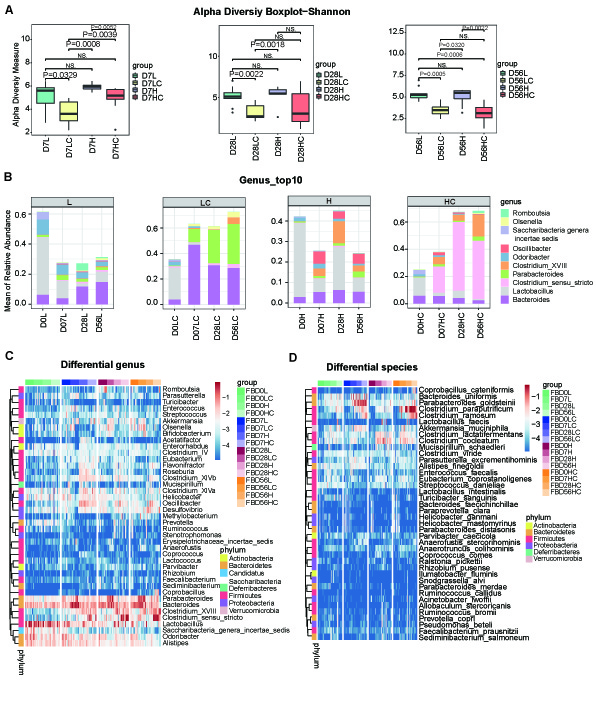


Supplementary Figure 3. Data analysis of intestinal microbiota. A. Shannon index analysis of intestinal microbiome over time; B. Top 10 dominant genera of intestinal flora in each group over time; C. Heat map of different genera at the same point in different groups; D. Heatmap of differential fecal species at the same time points.

Supplementary Figure 4


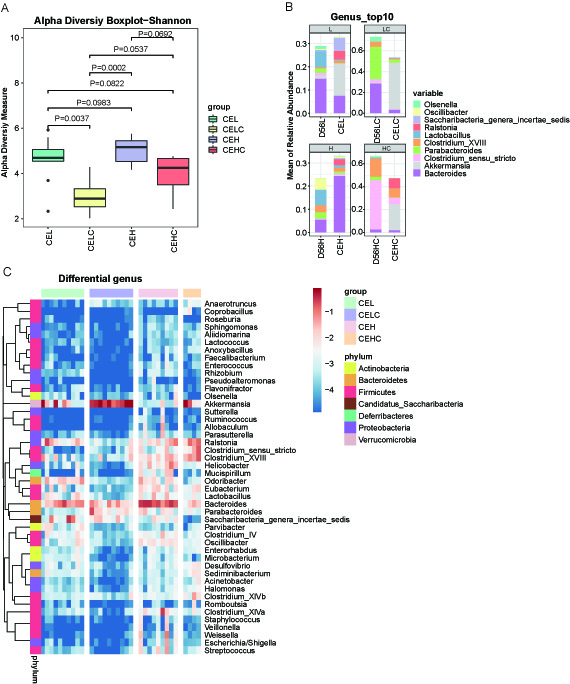


Supplementary Figure 4. The effects of COS on cecal microbiota. A. α-diversity of ceacal microbiota; B. The compositions of mouse ceacal bacteria at the genus level; C. Heatmap of differential cecum genera at the same time point.

Supplementary Table 1. Energy contained in foods.

| Product # | D12492-5%cellulose | | D12492-5%COS | | D12450J-5%cellulose | | D12450J-5%COS | |
| --- | --- | --- | --- | --- | --- | --- | --- | --- |
|  | gm% | kcal% | gm% | kcal% | gm% | kcal% | gm% | kcal% |
| Protein | 26.6 | 20 | 26.6 | 20 | 19.2 | 20 | 19.2 | 20 |
| Carbohydrate | 30.4 | 20 | 30.4 | 20 | 71.2 | 70 | 71.2 | 70 |
| Fat | 35.4 | 60 | 35.4 | 60 | 4.3 | 10 | 4.3 | 10 |
| Total |  | 100 |  | 100 |  | 100 |  | 100 |
| kcal/gm | 5.32 |  | 5.32 |  | 3.83 |  | 3.83 |  |
|  |  |  |  |  |  |  |  |  |
| Ingredient | gm | kcal | gm | kcal | gm | kcal | gm | kcal |
| Casein, 30 Mesh | 200 | 800 | 200 | 800 | 200 | 800 | 200 | 800 |
| L-Cystine | 3 | 12 | 3 | 12 | 3 | 12 | 3 | 12 |
| Corn Starch | 0 | 0 | 0 | 0 | 506.2 | 2024.8 | 506.2 | 2024.8 |
| Maltodextrin 10 | 125 | 500 | 125 | 500 | 125 | 500 | 125 | 500 |
| Sucrose | 68.8 | 275 | 68.8 | 275 | 68.8 | 275.2 | 68.8 | 275.2 |
| Cellulose, BW200 | 38.1 | 0 | 15.2 | 0 | 52.9 | 0 | 21.2 | 0 |
| Chitosanrz | 0 | 0 | 22.9 | 0 | 0 | 0 | 31.7 | 0 |
| Soybean Oil | 25 | 225 | 25 | 225 | 25 | 225 | 25 | 225 |
| Lard | 245 | 2205 | 245 | 2205 | 20 | 180 | 20 | 180 |
| Mineral Mix S10026 | 10 | 0 | 10 | 0 | 10 | 0 | 10 | 0 |
| DiCalcium Phosphate | 13 | 0 | 13 | 0 | 13 | 0 | 13 | 0 |
| Calcium Carbonate | 5.5 | 0 | 5.5 | 0 | 5.5 | 0 | 5.5 | 0 |
| Potassium Citrate, 1 H2O | 16.5 | 0 | 16.5 | 0 | 16.5 | 0 | 16.5 | 0 |
| Vitamin Mix V10001 | 10 | 40 | 10 | 40 | 10 | 40 | 10 | 40 |
| Choline Bitartrate | 2 | 0 | 2 | 0 | 2 | 0 | 2 | 0 |
| Total | 761.95 | 4057 | 761.95 | 4057 | 1057.95 | 4057 | 1057.95 | 4057 |

Supplementary Table 2. Primer sequences used for qRT-PCR

| Gene | Primer sequences |
| --- | --- |
| GAPDH | F5 -AAATGGTGAAGGTCGGTGTG-3 |
|  | R5-TGAAGGGGTCGTTGATGG-3 |
| TNF-α | F5 -GTCCGGGCAGGTCTACTTTG-3 |
|  | R5-GGGGCTCTGAGGAGTAGACA-3 |
| IL-6 | F5 –CTGGTCTTCTGGAGTACCATAGC-3 |
|  | R5-TCTGTGACTCCAGCTTATCTCTTGG-3 |
| IL-10 | F5-GGGCCCTTTGCTATGGTGT-3 |
|  | R5-GGGGATGACAGTAGGGGAAC-3 |
| IL-1b | F5-AATGCCACCTTTTGACAGTGATG-3 |
|  | R5-ATGTGCTGCTGCGAGATTTG-3 |
| IFN-γ | F5-GCTACACACTGCATCTTGGC-3 |
|  | R5-GCTTTCAATGACTGTGCCGT-3 |
| MCP-1 | F5-ACCACCTCAAGCACTTCTGT-3 |
|  | R5-TAAGGCATCACAGTCCGAGT-3 |
| ACC1 | F5-GGAGATGTACGCTGACCGAG-3 |
|  | R5-TACCCGACGCATGGTTTTCA-3 |
| Fas | F5-CTGCACCCTGACCCAGAATA-3 |
|  | R5-AGTGTTCACAGCCAGGAGAA-3 |
| Glut | F5-AAGCCATCATGCGATTGGTC-3 |
|  | R5-GCAGCAACCCTCCAATGAAA-3 |
| PAI-1 | F5-AGCTTTGTGAAGGAGGACCG-3 |
|  | R5-CTGATGGGCTGTGTGGGATT-3 |
| TJP | F5-TGAACGTCCCTGACCTTTCG-3 |
|  | R5-CTGTGGAGACTGCGTGGAAT-3 |
| occludin | F5-CCACCCCCATCTGACTATGC-3 |
|  | R5-TCGCTTGCCATTCACTTTGC-3 |
| SREBP1 | F5-AGCAGTCACCAGCTTCAGTC-3 |
|  | R5-GGTCATGTTGGAAACCACGC-3 |
| Ifng | F5-GCTACACACTGCATCTTGGC-3 |
|  | R5-GCTTTCAATGACTGTGCCGT-3 |
| Leptin | F5-GGCTTCACCCCATTCTGAGT-3 |
|  | R5-ACATTTTGGGAAGGCAGGCT-3 |
| Insulin | F5-TTCAGACCTTGGCGTTGGAG-3 |
|  | R5-GGTCGAGGTGGGCCTTAGTT-3 |
| Glut4(Slc2a4) | F5-GCCCCATTCCCTGGTTCATT-3 |
|  | R5-GACCCATAGCATCCGCAACA-3 |
